# Surprisingly facile CO_2_ insertion into cobalt alkoxide bonds: A theoretical investigation

**DOI:** 10.3762/bjoc.11.144

**Published:** 2015-07-31

**Authors:** Willem K Offermans, Claudia Bizzarri, Walter Leitner, Thomas E Müller

**Affiliations:** 1 CAT Catalytic Center, RWTH Aachen University, Worringerweg 2, 52074 Aachen, Germany; 2 Lehrstuhl für Technische Chemie und Petrolchemie, ITMC, RWTH Aachen University, Worringerweg 1, D-52074 Aachen, Germany

**Keywords:** activation, alkoxide, carbon dioxide, cobalt, insertion, salen

## Abstract

Exploiting carbon dioxide as co-monomer with epoxides in the production of polycarbonates is economically highly attractive. More effective catalysts for this reaction are intensively being sought. To promote better understanding of the catalytic pathways, this study uses density functional theory calculations to elucidate the reaction step of CO_2_ insertion into cobalt(III)–alkoxide bonds, which is also the central step of metal catalysed carboxylation reactions. It was found that CO_2_ insertion into the cobalt(III)–alkoxide bond of [(2-hydroxyethoxy)Co^III^(salen)(L)] complexes (salen = *N*,*N*”-bis(salicyliden-1,6-diaminophenyl)) is exothermic, whereby the exothermicity depends on the *trans*-ligand L. The more electron-donating this ligand is, the more exothermic the insertion step is. Interestingly, we found that the activation barrier decreases with increasing exothermicity of the CO_2_ insertion. Hereby, a linear Brønsted–Evans–Polanyi relationship was found between the activation energy and the reaction energy.

## Introduction

Carbon dioxide (CO_2_) has been known to be an attractive carbon source for decades [1–6]. Even so, industrial processes using CO_2_ as chemical feedstock are limited to the production of few large scale chemicals such as urea, methanol, salicylic acid as well as inorganic and organic carbonates [5]. Since CO_2_ is captured in huge amounts from the flue gases of fossil fuel combustion, it would be very beneficial to harness a part of this stream for producing valuable products [7]. From a thermodynamic point of view, however, CO_2_ is highly stable and, thus, shows low reactivity. One way to overcome this thermodynamic hurdle is to react carbon dioxide with relatively high-energy molecules such as ammonia, hydrogen, epoxides or lactones [8]. Nevertheless, the reactions frequently involve a substantial kinetic barrier or are accompanied by side reactions that, so far, have resulted in insufficient yields and/or selectivities. Even though there are numerous compounds that might react with CO_2_, active and selective catalysts for these reactions are scarce [9].

**Scheme 1:**
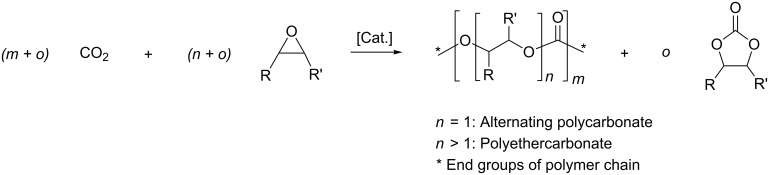
Reaction of carbon dioxide with epoxide to yield alternating polycarbonates, polyethercarbonates or cyclic carbonates.

The reaction of CO_2_ with epoxides yields alternating polycarbonates, polyethercarbonates or cyclic carbonates (Scheme 1). The production of CO_2_-based polymers is considerably more challenging compared to the formation of cyclic carbonates. In consequence, industrially relevant catalysts, combined with efficient processes, have only recently emerged for the manufacture of alternating polycarbonates [10] and polyethercarbonates [11]. The respective research has mainly focussed on homogeneous zinc–alkoxide complexes [12] and chromium– [13–18] and cobalt–salen complexes [19–22] and heterogeneous double metal cyanide (DMC) catalysts [11,23–26]. In comparison, industrially well-established catalysts are available to accelerate the production of cyclic carbonates [27–29]. As the CO_2_-based polymers are thermodynamically less stable than cyclic carbonates, a kinetic control must be attained to direct the reaction to the polymeric products. To selectively lower the activation barrier towards polycarbonates, a rational development of suitable catalysts is essential. This current study aims to elucidate the reaction step of CO_2_ insertion, catalysed by cobalt(III)–salen complexes, using density functional theory calculations (DFT).

## Results and Discussion

### Background research: Formation of CO_2_-based polymers

The catalytic pathways for producing polycarbonate and polyethercarbonates have been explored in several studies.

**Scheme 2:**
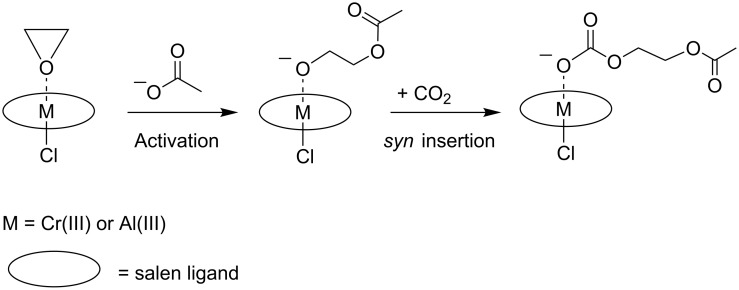
Epoxide and CO_2_ copolymerisation by homogeneous Cr(III)– and Al(III)–salen complexes.

Rieger et al. studied the mechanisms of the copolymerisation by homogeneous chromium(III)– and aluminium(III)–salen complexes and by heterogeneous zinc-dicarboxylates [30–31]. Experimental work on the chromium(III)– and aluminium(III)–salen complexes was combined with a theoretical study, in which the initiation and propagation steps of the copolymerisation were addressed. The initiation started with the coordination of epoxide at a penta-coordinated metal–salen complex, followed by a nucleophilic, S_N_2-like, back-side attack on such a coordinated epoxide. The propagation comprised a *syn*-insertion of CO_2_ into the metal–alkoxide bond and a bimolecular chain transfer of a metal-bound, carbonate-terminated, growing chain to a metal-coordinated and, thus, activated epoxide (see Scheme 2). For hepta-coordinated Cr(III)– and Al(III)–salen complexes, the CO_2_ insertion requires considerable energy, and it was concluded to be the rate-determining step especially at low CO_2_ concentrations.

A mechanism for the copolymerisation of epoxides and CO_2_, catalysed by cobalt(III)–salen complexes was proposed by Lu et al. [32]. The authors stated that a general mechanism with pending (re)attachment of growing carbonate chains or units might explain the stability, activity and selectivity of the cobalt(III)–salen complexes. Even though CO_2_ insertion plays a key role in this mechanism, it is only schematically depicted.

**Figure 1:**
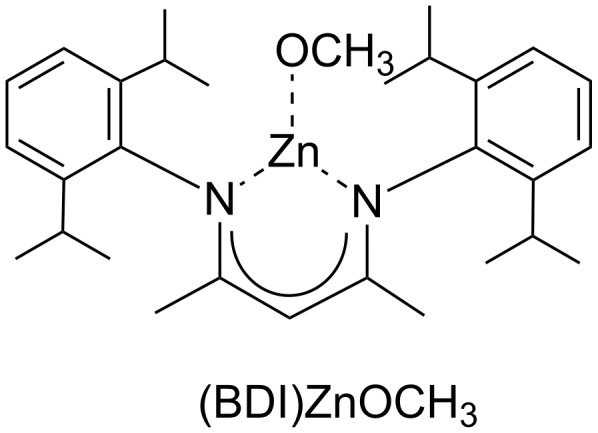
The tri-coordinated di-iminate zinc–alkoxide complex [(BDI)ZnOCH_3_].

The mechanism of the Zn(II)-catalysed copolymerisation of CO_2_ and cyclohexene oxide was the subject of a molecular orbital study on the tri-coordinated di-iminate zinc–alkoxide complex [(BDI)ZnOCH_3_], whereby BDI is *N*-(2,6-iPr_2_C_6_H_3_C(Me)-CHC(Me)-*N*-(2,6-iPr_2_C_6_H_3_) (see Figure 1) [33]. The study was based on the ONIOM [34–35] method, which is a computationally intricate method applied to atoms directly involved or in close vicinity of the reaction center, but involves less demanding calculations for the outer periphery.

Though kinetically favoured, the insertion of CO_2_ into the zinc–alkoxide bond turned out to be thermodynamically less favourable than the insertion of epoxide. A low activation barrier found for the CO_2_ insertion into the zinc–alkoxide bond may be related to the use of a relatively high energy unsaturated 16-electron complex. In the reaction mixture of neat cyclohexene oxide, the complex will readily coordinate additional cyclohexene oxide as solvent molecule(s). Accompanied by high activation barriers, the ring opening of the epoxide and the electrophilic insertion into either the zinc–alkoxide or zinc carbonate bond occur consecutively. Only if the ring is pre-activated by, for example, sufficient ring strain, the polymerisation reaction does become feasible.

**Scheme 3:**
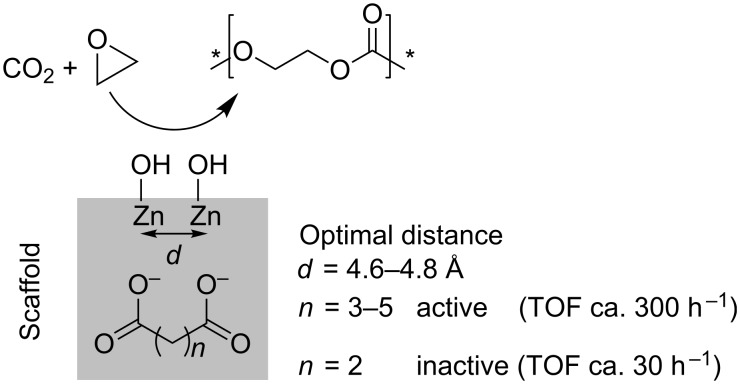
Heterogeneous zinc dicarboxylates for the copolymerisation of CO_2_ and epoxides. (* = End group of polymer chain).

In mechanistic studies on zinc dicarboxylates and their application in the heterogeneously catalysed copolymerisation of CO_2_ and epoxides a bimetallic mechanism was proposed [31]. As post-modification of the prepared catalysts with water proved to be important for the activity of the catalysts, the presence of ZnOH groups at the surface may be inevitable for the catalysis. Two zinc atoms, in a distance of approximately 4.6–4.8 Å, are present at the surfaces of zinc glutarate, zinc adipate and zinc pimelate (Scheme 3). This spatial conformation of two zinc atoms is not present on the surface of zinc succinate. This was used to explain the activity of the former three and the inactivity of the latter. Theoretical calculations revealed a decrease in the activation barrier for the homopolymerisation as well as the copolymerisation step with increasing metal–metal distance. Moreover, the difference in activation barriers between the homo- and copolymerisation decreased with an increase in the metal–metal distance, but levelled at distances above 5 Å. Thus, a distance of 4.6–4.8 Å is a good compromise to avoid the homopolymerisation and to enhance the catalytic activity for the copolymerisation. Involving two proximate zinc atoms, which coordinate the reactants, the ring-opening of epoxides by alkylcarbonates or alkoxides occurs in a bimetallic fashion [31,36]. The insertion of CO_2_ was not further addressed and was regarded as facile by the authors.

### Background research: Formation of cyclic carbonates

**Scheme 4:**
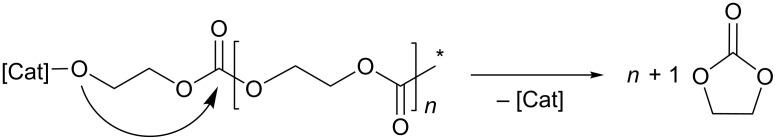
Backbiting mechanism for the formation of cyclic carbonates.

Cyclic carbonates can be formed either by a backbiting mechanism from a growing polymer chain [30] (Scheme 4) or directly by the catalytic cycloaddition of CO_2_ and epoxides [27–29].

**Scheme 5:**
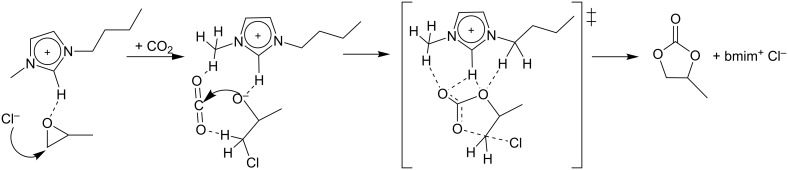
Two-step pathway for the cycloaddition of propylene oxide and CO_2_ in the ionic liquid 1-butyl-3-methylimidazolium chloride (bmim^+^ Cl^−^).

Sun and Zhang modelled the cycloaddition of CO_2_ with propylene oxide, catalysed by alkylmethylimidazolium chlorine ionic liquids [37]. They identified competitive three-step and two-step pathways, both having the ring opening of propylene oxide as the rate-determining step. In the three-step pathway, the ring opening precedes the CO_2_ insertion. By contrast, in the two-step pathway, the ring opening and CO_2_ insertion occur simultaneously, whereby at a given time three molecules need to orientate themselves into a particular configuration to form the transition state (see Scheme 5). This is demanding with respect to the entropy effects. In both pathways, the CO_2_ addition to the epoxide was found to be facilitated considerably by hydrogen bonding interactions. Thus, a scaffold of hydrogen bonds can compensate for the lack of a Lewis acid metal center to activate the CO_2_ molecule.

**Scheme 6:**
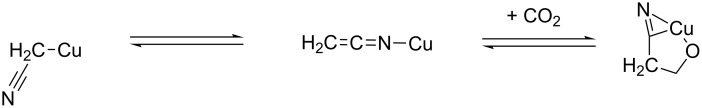
Formation of copper(I) cyanoacetate for the activation of CO_2_.

In addition, Zhang et al. investigated the coupling of propylene oxide with CO_2_ catalysed by a copper(I)–cyanomethyl complex [38]. They proposed two reaction steps: insertion of carbon dioxide forming a copper(I) cyanoacetate as activated CO_2_ carrier that reacts in a second step with propylene oxide (see Scheme 6). Formation of propylene carbonate subsequently involves the oxidative transformation of an eight-membered ring intermediate to a six-membered ring.

**Scheme 7:**
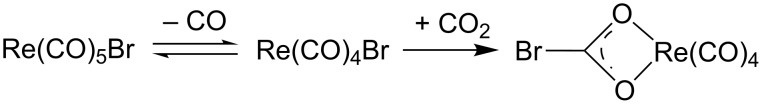
Activation of CO_2_ by nucleophilic attack of bromide in the Re(I)-catalysed cycloaddition.

Moreover, Wu et al. studied a) the cycloaddition of ethylene oxide and CO_2_, catalysed by Ni(PPh_3_)_2_ [39], b) the cycloaddition of chloromethyloxirane and CO_2_, catalysed by Re(CO)_5_Br [40] and c) the cycloaddition of 4-(phenoxymethyl)-1,3-dioxolan-2-one and CO_2_, catalysed by LiBr [41]. The preferred pathways principally involve ring opening of the epoxide, followed by CO_2_ insertion and ring closure of the cyclic carbonate. Interestingly, in the Re(I)-catalysed reaction (b), an alternative reaction pathway was followed, whereby the first step is CO_2_ insertion, induced by the nucleophilic attack of the bromide on the carbon atom of CO_2_. The transition state comprised a four membered oxametallacycle (see Scheme 7). The alternative insertion of epoxide into the Re–Br bond was found to be significantly more energy demanding.

### Background research: Activation of CO_2_ by insertion into metal–H and metal–R bonds

**Scheme 8:**
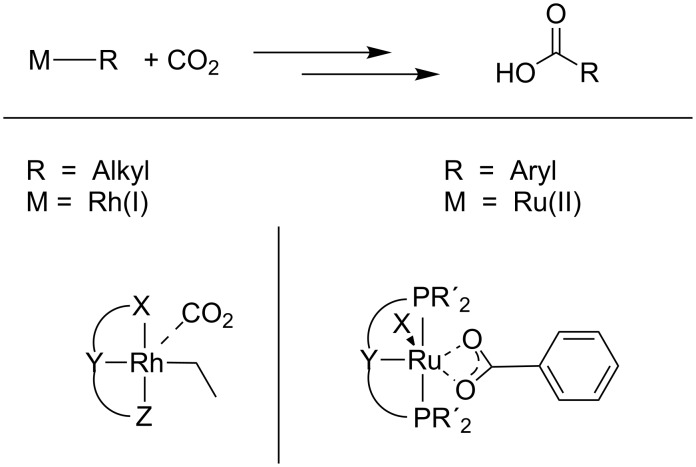
Direct catalytic carboxylation of aliphatic compounds and arenes by rhodium(I)– and ruthenium(II)–pincer complexes, respectively.

The insertion of CO_2_ into metal–alkoxide bonds is believed to be a key step in the aforementioned catalytic routes to polycarbonates and cyclic carbonates. In this context, several studies on the activation of CO_2_ and insertion into metal–H and metal–X bonds have been reported [42–43] (see Scheme 8).

**Scheme 9:**
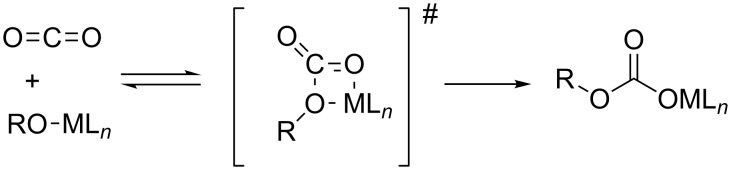
Insertion of carbon dioxide into a metal–oxygen bond via a cyclic four-membered transition state. R is either an aliphatic or aromatic group.

The reaction of CO_2_ with metal alkoxides is reversible as the metal–oxygen bond strengths of metal alkoxides and of metal carbonates are similar [44–45]. The theoretically derived mechanism points out an intermediate encounter complex between CO_2_ and metal alkoxide. Formation of this associative complex is endergonic due to the entropy effects [45]. The transition state for the CO_2_ insertion into metal–oxygen bonds has been postulated to be a four-membered ring consisting of the metal, the carbon and two oxygen atoms (Scheme 9) [44]. For this cycloaddition pathway, a vacant coordination site at the metal center is not necessary.

**Scheme 10:**
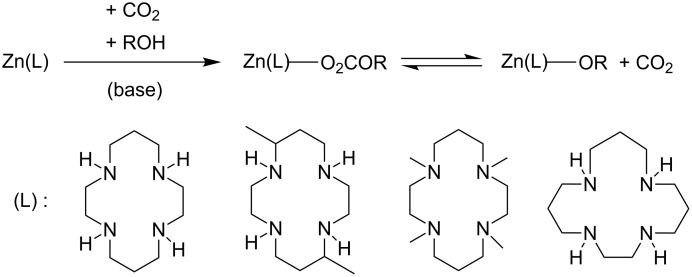
Facile CO_2_ uptake by zinc(II)–tetraazacycloalkanes.

Kato et al. reported on the facile uptake of CO_2_ into zinc–alkoxide bonds leading to zinc-coordinated monoalkyl carbonates by zinc(II)–tetraazacycloalkane complexes (Scheme 10) in the presence of alcohols [46]. Here, the uptake of CO_2_ from the air was found to occur spontaneously, even in neutral solution and low temperatures below 10 °C. This clearly exemplifies the high reactivity of the CO_2_ molecule under appropriately chosen conditions.

Later, Kunert et al. investigated the mechanism of CO_2_ insertion into zinc(II)-phenoxide to form zinc(II)–phenylcarbonate [47]. The CO_2_ insertion was found to take place via electrostatic interactions between the electron lone pairs of the zinc-bonded oxygen and the carbon of CO_2_, forming an intermediate that finally produces a stable insertion product.

### Current research: CO_2_ Insertion into cobalt–alkoxide bonds

Even though CO_2_ insertion into metal–oxygen bonds plays a key role in the mechanistic pathways for the copolymerisation of epoxides and CO_2_, this elementary step is only schematically depicted. Consequently, there is still a lack of understanding of the CO_2_-insertion step on a molecular or atomic level. In particular, a detailed study of CO_2_ insertion into cobalt–alkoxide bonds is still missing.

**Figure 2:**
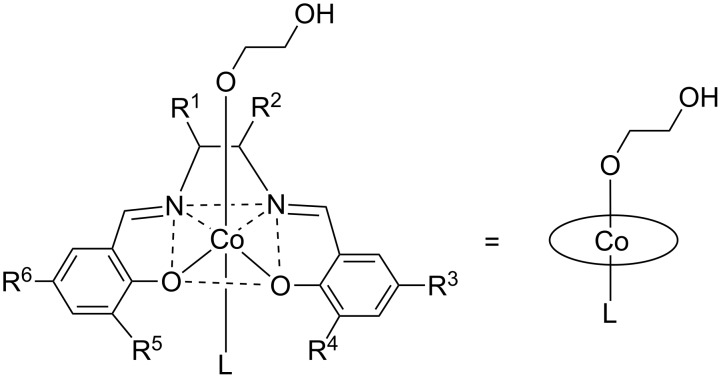
The [(2-hydroxyethoxy)Co^III^(salen)(L)] complex chosen as catalyst model for the calculations; **1**: R^1–6^ = H; **1a**: 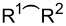 = -C_4_H_8_-, R^3–6^ = H; **1b**: 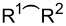 = -C_4_H_8_-, R^3–6^ = *t*-Bu.

This present work aims to investigate CO_2_ activation, in general, and the insertion step in the copolymerisation of CO_2_ and epoxides, in particular, by DFT methods, using the cobalt(III)–salen complex illustrated in Figure 2 as a model for the catalyst. For the sake of simplicity, the salen ligand is depicted as a circle, although in the discussed calculations the salen ligand is represented by its correct nuclei and electrons. For most calculations the substituents were taken as protons (R^1–6^ = H, salen ligand **1**). To test the validity of the model with respect to the relative energies and geometries, the salen ligand sphere was extended to a cyclohexyl backbone (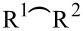 = -C_4_H_8_-) with the substituents R^3–6^ = H (salen ligand **1a**) and R^3–6^ = *t*-Bu (salen ligand **1b**). The ligand L was varied in the calculations from stronger electron-donating nucleophiles, such as chloride and acetate, to weaker electron-donating ligands, such as 2,4-dinitrophenolate.

**Figure 3:**
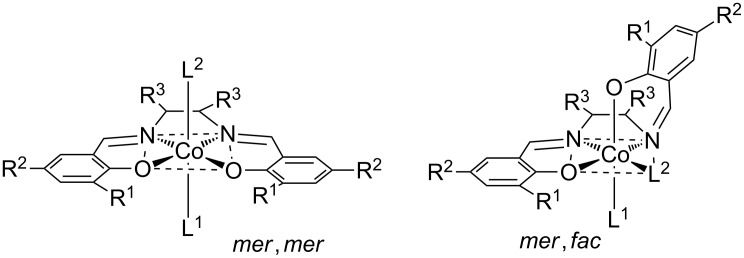
The two most relevant configurations of [(2-hydroxyethoxy)Co^III^(salen)(L)] complexes. The left-hand model shows a *mer*,*mer*-configuration, while the right-hand models shows a *mer*,*fac*-configuration of the salen ligand.

The salen ligand can assume different configurations relative to the cobalt center [48]. To test the significance of the *mer*,*mer*- and the *mer*,*fac*-configuration (Figure 3), the total energy was calculated. It was found that the *mer*,*mer*-configuration was significantly more stable than the *mer*,*fac*-configuration. The difference in total energy was 96 kJ·mol^−1^ for chloride and 30 kJ·mol^−1^ for 2,4-dinitrophenolate. Therefore, the further study was limited to models with *mer*,*mer*-configuration. Here, the salen ligand coordinates the central cobalt atom in a square-planar fashion, whereby the *trans*-ligand L is located in the lower and 2-hydroxyethoxide in the upper hemisphere, respectively.

**Figure 4:**
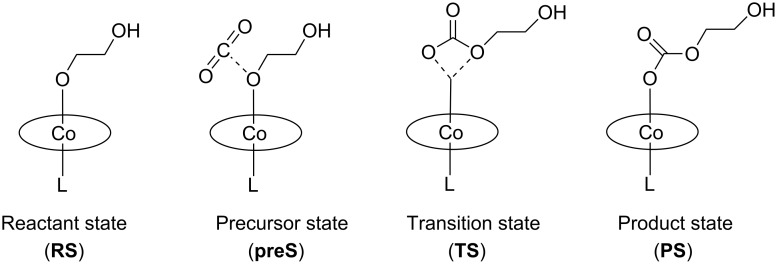
Carbon dioxide insertion into the cobalt(III)–alkoxide bond of [(2-hydroxyethoxy)Co^III^(salen)(L)] complexes.

The modelling of the insertion reaction started with the geometry optimization of a free CO_2_ molecule and the parent [(2-hydroxyethoxy)Co^III^(salen)(L)] complex. This is indicated as the reactant state (**RS**) in Figure 4. We found a linear CO_2_ molecule (179.998°) with a bond distance of 1.177 Å, which is in good agreement with experimental and theoretical data [37]. In the [(2-hydroxyethoxy)Co^III^(salen)(L)] complex, the cobalt atom was located at the center of the salen ligand. The distances between the cobalt center and the nitrogen and oxygen atoms of the salen ligand were 1.92 Å and 1.94 Å, respectively. This is consistent with the single crystal solid-state structure reported for [Co^III^(salen)(dinitrophenolate)] [49]. The distance between cobalt and the oxygen atom of the alkoxide chain was 1.9–1.94 Å, with a small yet systematic variation in dependence on the nucleophilicity of L. The more nucleophilic L was, the longer the Co–OR distance was. The Co–L bond length was calculated to be 2.03–2.13 Å for L with a coordinating oxygen-atom and 2.35 Å for chloride. The calculated Mulliken charge was 0.7–0.72 C on the cobalt center for L with a coordinating oxygen atom and 0.64 C for chloride consistent with a more nucleophilic character of chloride.

**Table 1:** Relative energies with respect to free CO_2_ and free cobalt(III)–salen complex of the precursor, transition and product state of the CO_2_ insertion reaction as shown in Figure 4.

Salen ligand	*trans*-Ligand	Precursor state^a^ (**preS**)	Transition state^a^ (**TS**)	Product state^a^ (**PS**)	Activation barrier	Reaction energy

		[kJ·mol^−1^]	[kJ·mol^−1^]	[kJ·mol^−1^]	[kJ·mol^−1^]	[kJ·mol^−1^]

**1**	chloride	−14	31	−47	45	−33
**1**	CH_3_C(O)O^−^	−14	45	−30	59	−16
**1a**^b^	CH_3_C(O)O^−^	−12	41	−31	53	−19
**1b**^c^	CH_3_C(O)O^−^	−11	39	−37	50	−26
**1**	*p*-methoxyphenolate	−14	41	−33	55	−19
**1**	CCl_3_C(O)O^−^	−21	34	−30	55	−9
**1**	2,4-dinitrophenolate	−22	68	−29	90	−7
**1**	2,4,6-trinitrophenolate	−11	79	−16	90	−5
**1**	TBD^d^	−14	65	−29	79	−15
**1**	none	−13	58	−6	71	7
**1**	ethylene oxide	−17	100	−8	117	9

^a^Potential energies relative to free CO_2_ and the free cobalt(III)–salen complex, i.e., the reactant state was set to zero for each *trans*-ligand L. ^b^Salen with cyclohexyl backbone, 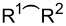 = -C_4_H_8_-, R^3–6^ = H ^c^Salen with cyclohexyl backbone, 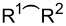 = -C_4_H_8_-, R^3–6^ = *t*-Bu ^d^TBD = 1,5,7-triazabicyclo[4.4.0]dec-5-ene.

In a search for possible interactions between CO_2_ and the cobalt-salen-alkoxide complex, the calculations revealed a precursor state, formed during the approach of CO_2_ to the [(2-hydroxyethoxy)Co^III^(salen)(L)] complex (Figure 4). Inspection of the geometry revealed that the closest intermolecular distance was between the carbon atom of CO_2_ and the oxygen atom of the alkoxide chain; the shortest distance was for chloride (1.67 Å) and the longest for a pending 1,5,7-triazabicyclo[4.4.0]dec-5-ene group (TBD) as ligand L (3.59 Å, see Supporting Information File 1). The change in the CO_2_ bond angle with respect to linear CO_2_ was marginal (2° to 6°). For the chloro complex [(2-hydroxyethoxy)Co^III^(salen)(Cl)], the potential energy surface was found to be extremely flat with respect to the O–CO_2_ distance, meaning that minimum states with a 40° bent CO_2_ molecule and states with a practically linear CO_2_ molecule have almost equal energy. We noticed a similar configuration of the four atoms in the transition state (vide infra). The geometry in the cobalt–salen complexes was very similar to the reactant state; solely the cobalt–alkoxide bond length increased slightly yet systematically. For each L, the precursor state (**preS**) was 10–20 kJ·mol^−1^ more stable than the reactant state (**RS**). Thus, the change in energy was small with respect to the reactant state. Consistent with an associative step, the entropy change was negative and the change in free energy was positive. Consistent with the weak interaction between CO_2_ and the [(2-hydroxyethoxy)Co^III^(salen)(L)] complex, the calculated Mulliken charge on the metal center and its surrounding geometry hardly changed with respect to the reactant state. Similar precursor states were reported for a supramolecular complex of ethylmethylimidazolium chloride, CO_2_, and propylene oxide (complex c in [37]) and for the association complex of [Ni(OH)(pincer ligand)] and CO_2_ [50].

The calculations for the product state (**PS**) revealed an optimized structure for each ligand L, whereby the carbonate chain was coordinated in a mono-dentate fashion to the cobalt center (Figure S1 in Supporting Information File 1). The calculated Co^III^–L bond lengths were 1.95–2.08 Å, when the coordinating atom was oxygen, and 2.29 Å, when it was chloride. In general, these bond lengths were shorter than in **RS** (by 0.03–0.06 Å). Independently of the choice of L, the cobalt atom was located in the center of the salen ligand at an average distance of 1.93(2) Å from the coordinating atoms. Thus, there was only a minor geometrical change in respect to the lower hemisphere of the [(2-hydroxyethoxy)Co^III^(salen)(L)] complex. The distance of the newly formed Co^III^–O bond varied from 1.99 Å to 1.93 Å depending on the nucleophilicity of L: the less nucleophilic the ligand L was, the shorter the Co^III^–O bond was. The carbon–oxygen bond lengths within the carbonate unit were 1.294(5) Å for C1–O3, 1.236(3) Å for C1–O4, and 1.392(11) Å for C1–O5, with only little variation in length upon variation of L. Hence, the carbon–oxygen bond lengths were clearly lengthened relative to the CO_2_ molecule. The energies of the product states were lowered with respect to the reactant states. The less nucleophilic the ligand L was, the less stabilized the carbonate species was with respect to free CO_2_ and the [(2-hydroxyethoxy)Co^III^(salen)(L)] complex. The respective Mulliken charge on the cobalt atom was 0.71(1) C in the case of L having an oxygen-coordinating atom and 0.62 C for L being chloride. Consequently, there was no change in the charge of cobalt with respect to the **RS**.

Using the precursor and product states as the anchor points, the transition states were located for each L. All determined transition states were geometrically similar, depicting a cyclic arrangement around the cobalt(III) atom, an oxygen and carbon atom of CO_2_ and the oxygen atom of the original Co–O alkoxide bond (Figure 4). These configurations were identical to the arrangement of the reactive atoms in the transition states of the reaction between [Ni(OH)(pincer ligand)] complexes and CO_2_ [50]. Since the configurations of the reactive atoms are essentially identical in the reactant, precursor, transition and product states, we conclude that the mechanism of the CO_2_ insertion reaction is very similar for cobalt-alkoxide and nickel-hydroxide complexes.

In the case of Cr(III)–salen complexes, it was found [18] that in the transition state of the CO_2_ insertion step, the Cr–O bond is elongated (from 1.96 Å to 2.12 Å) as well as the CO_2_ molecule is bent (OCO angle 146.7°). The new C–O and Cr–O bonds (after CO_2_ insertion) form synchronously, without prior activation by binding to the Lewis acidic Cr(III) center, as the coordination sphere of chromium is saturated.

Regarding Al(III)–salen complexes, the insertion step into an Al(III)-alkoxide bond is a simple intramolecular process with a small activation energy (∆*E*^#^ = 9.12 kcal/mol) [30,51]. Nevertheless, a bimetallic aluminium complex is considered in the mechanism. Since it is a bimolecular reaction, the Gibbs energies will be higher. Carbon dioxide insertion may well be the rate-determining step, certainly at lower CO_2_ pressure.

Also in the transition state (**TS**) the cobalt atom was located at the center of the coordination sphere at a distance of 1.9 Å from the salen ligand. Thus, there is no significant movement of the cobalt center during the reaction with CO_2_. The Co^III^–L bond lengths were calculated to be 1.90–1.98 Å for the coordinating atom being oxygen and 2.24 Å for chloride. The Co^III^–L bond distances were found to shorten from **RS** to **preS** and **TS**. Although this bond elongates from **TS** to **PS**, the length is still shorter than in **RS**. As these systematic changes were only in the 0.01–0.10 Å range, the geometrical changes in the lower hemisphere of the cobalt(III)–salen complex are only minor during the CO_2_ insertion. This is consistent with the changes of the calculated Mulliken charge on the cobalt center. The charge at the transition state was 0.7–0.73 C for L with an oxygen-coordinating atom and was 0.6 C for chloride. The changes over the different states were only noticeable on the scale of 0.01–0.1 C.

Focussing on the arrangement of the four reactive atoms in the transition state and, in particular, on the forming carbonate unit, it is noteworthy that the distance between the carbon atom of CO_2_ and the oxygen atom of the alkoxide chain was only slightly larger in the transition state than in the product state. This applies to the carbon–oxygen bond length of the carbonyl group as well. By contrast, the bond length between the carbon atom of CO_2_ and the oxygen atom of the forming Co–O–C moiety was slightly shorter in the transition state than in the product state. Although the changes in the bond lengths of the forming carbonate unit were systematic for all L, the changes were small (0.01–0.05 Å). This is consistent with the minor changes in the (O–C–O) angle of the forming carbonate unit between **TS** and **PS**. This means that the configuration of the carbonate unit in the transition state is already very close to the one in the product state. Hence, the major geometrical rearrangement from the transition to product state is concentrated in the O–Co–O configuration, i.e., in the change of the hapticity of the cobalt center from η^2^ to η^1^ and the inherent changes in bond lengths. The cobalt(III)–alkoxide bond breaks and the newly formed Co^III^–O bond is shortened by ca. 0.8–1.4 Å in the switchover from **TS** to **PS**. Particular attention ought to be given to the cobalt–oxygen bond lengths of the different L = CH_3_C(O)O^−^ models. In the transition state, there was no difference between the minimal model (salen ligand **1**) and the extended model with only the cyclohexyl backbone (salen ligand **1a**). However, the introduction of the additional *t*-Bu groups (salen ligand **1b**) led to a significant increase of ca. 0.5 Å in these lengths. This is remarkable, since the bond lengths and the respective relative energies in the reactant, precursor and product states were quite similar for all L = CH_3_C(O)O^−^ models. This means that the coordinated L = CH_3_C(O)O^−^ has no significant influence on the CO_2_ insertion itself, but only on the relative energy of the cobalt–salen complex. Since the variation in the cobalt–oxygen bond lengths of the O–Co^III^–O units over all nucleophiles L was relatively large, roughly 2.5–3.5 Å for both bonds, and not systematic for the various ligands L, we assume that this reduced influence of the nucleophile L on the CO_2_ insertion applies to the other ligands L as well. This is also consistent with the minor changes in the geometry of the lower hemisphere of the [(2-hydroxyethoxy)Co^III^(salen)(L)] complexes during CO_2_ incorporation and in the Mulliken charge on the cobalt center.

### Current research: Brønsted–Evans–Polanyi relationship

**Figure 5:**
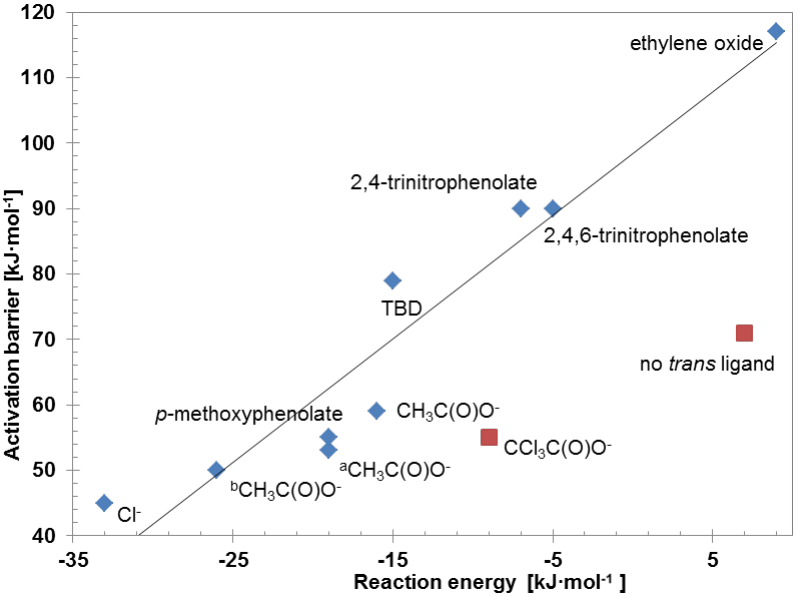
Energy relationship between the activation barrier and the reaction energy of the CO_2_ incorporation reaction. Plotted are the results for different nucleophiles L, attached to the Co^III^–salen base structure as depicted in Figure 2; ^a^salen ligand **1a**: 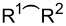 = -C_4_H_8_-, R^3–6^ = H; ^b^salen ligand **1b**: 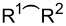 = -C_4_H_8_-, R^3–6^ = *t*-Bu. The energies calculated for those ligands depicted with a red square do not follow the BEP relationship (for details, see text). The line represents the equation *E*_a_ = (1.9 ± 0.2)·Δ*E* + (99 ± 4) [kJ·mol^−1^].

The relative energies of the precursor, transition and product states with respect to the reactant state are listed in Table 1. The energy difference between the product state and transition state relative to the reactant state in **preS** is the reaction energy and activation barrier, respectively. In Figure 5 the activation barrier is plotted as a function of the reaction energy. Clearly, the values of both energies depend on the choice of the nucleophile L. The more electron-donating the ligand is, the smaller the activation barrier is and the more exothermic the reaction energy is. This linear relationship between the activation barrier and reaction energy is an example of the Brønsted–Evans–Polanyi (BEP) relationship [52]. Mostly, the reaction energy is exothermic, which implies that the cobalt–carbonate bond is stronger than the cobalt–alkoxide bond. Since the alkoxide is a stronger Lewis base than the carbonate, we calculated an increase in bond strength between the carbonate unit and cobalt center with respect to the cobalt(III)–alkoxide bond, depending on the nucleophilicity of the ligand L. This corresponds to a decrease in the Lewis acidity of the cobalt center.

Consequently, the picture evolves to the reaction of an electrophilic CO_2_ molecule with a nucleophilic alkoxide chain connected to a cobalt(III) center. The CO_2_ molecule reacts via the carbon atom with the oxygen atom of the alkoxide chain to a carbonate unit. Here, the distance to the metal center does not seem to be energetically important, and the geometrical changes within the carbonate unit are similar for all considered *trans*-ligands L. Therefore, we assume that the significant energy differences in the activation and the reaction energy arise from the differing ability of the *trans*-ligand L to stabilize the cobalt(III)–salen complexes.

The energies calculated for L = CCl_3_C(O)O^−^ and for the absence of a *trans*-ligand do not follow the BEP relationship. This is readily explained by the electronic state and the HOMO–LUMO gap of these complexes [53]. Penta-coordinated cobalt(III)–salen complexes are high-spin complexes and spin-restricted calculations are insufficient to describe their electron configuration. In the hexa-coordinated [(2-hydroxyethoxy)Co^III^(salen)(trichloroacetate)] complex, the trichloroacetate ligand is so weakly coordinating that the same argument applies.

Thus, neither coordination of CO_2_ prior to the insertion of CO_2_ into a metal–X bond [50,53–54] nor activation of CO_2_ by a base (co-catalyst) is required. The base merely helps to open the epoxide, while CO_2_ being a weak electrophile, it requires a strong Lewis base in order for CO_2_ to react [54]. Addition of CO_2_ to the activated epoxide then occurs with a very low activation barrier [55]. In contrast, it has been generally believed up to now that, in the catalysed reaction of CO_2_ and epoxides, both reactants need to be activated [16,56].

## Conclusion

We found that CO_2_ readily inserts into the cobalt(III)–alkoxide bond of [(2-hydroxyethoxy)Co^III^(salen)(L)] complexes. Since the insertion occurs in a *syn* fashion via a four-membered ring structure, it does not need a free coordination site at the cobalt(III) center. We further found that the reaction energy of the CO_2_ insertion into the cobalt(III)–alkoxide bond is exothermic and that its magnitude depends on the *trans*-ligand coordinated to the cobalt(III)–salen complex. The more electron-donating this ligand is, the more exothermic the reaction energy is. Moreover, we found that the activation energy decreases with increasing exothermicity of the CO_2_ insertion. Thereby, a linear relationship between the activation energy and the reaction energy – a so-called Brønsted–Evans–Polanyi relationship – was found. This relationship enables one to predict the activation barrier from the reaction energy. Since the calculation of the former can be a delicate undertaking and the calculation of the latter is much easier, the relationship can be used to estimate the activation barrier for CO_2_ insertion into cobalt(III)–alkoxide bonds of similar, yet unknown complexes. Since we found a linear relationship between the reaction energy and activation energy and that the reaction is more exothermic for stronger electron-donating ligands, we logically conclude that the CO_2_ insertion is more facile for more electron-donating ligands. Since it was experimentally shown that cobalt(III)–salen complexes coordinated by electron-withdrawing ligands, are more active in the carbonate formation from epoxides and CO_2_, our findings indicate that the CO_2_ insertion cannot be the rate-determining step in these reactions.

## Experimental

Electronic structure calculations within the framework of density functional theory (DFT) were performed by using the DMol3 program [57–58]. The Perdew–Burke–Ernzerhof functional to account for the exchange correlation of the electrons was applied [59]. A double numerical basis set plus a polarization d-function on all non-hydrogen atoms and a polarization p-function on all hydrogen atoms was used to expand the one-electron Kohn–Sham eigenfunctions. If not otherwise stated, then full electron, spin-restricted calculations were performed. No constraints were applied during geometry optimisation. All reactant and product states were shown to be minima by a normal mode frequency analysis. The synchronous-transit method was applied to localize possible transition states [60]. These states were further optimized by following eigenvectors to gain the best predicted configuration of the transition state. Transition states were analysed by a normal mode frequency analysis and confirmed by only one imaginary frequency. It was confirmed by the calculation of the minimum energy path that the transition state connected the reactant and product state on the potential energy surface. Tests on several molecules showed that the electronic configuration can be trustfully determined by spin-restricted calculations. This is consistent with diamagnetic, low-spin, hexa-coordinated cobalt(III)–salen complexes, observed by Kemper et al. [61], and the results of DFT calculations of similar, hexa-coordinated cobalt(III)–salen complexes by Sun et al. [62]. In the spin-unrestricted calculations, a triplet state was considered and the minima of the corresponding spin-restricted calculations were used as initial structures. It turned out that the total energy of the geometrical optimised structures with triplet state was higher than the total energy of the spin-restricted calculations. A geometrical change was observed as well. The hexa-coordinated cobalt(III)–salen complex changed into a penta-coordinated, quadratic pyramidal structure. This is consistent with the observations and calculations of Kemper et al. and confirms that paramagnetism and penta-coordination of cobalt(III)–salen complexes are related. Solvent effects were not considered in the present study.

## Supporting Information

File 1 Atomic coordinates, calculated bond lengths and bond angles as well as calculated Mulliken charges for the reactant state, the precursor state, the transition state and the product state.
