# Supplementary material for: Surprisingly facile CO2 insertion into cobalt alkoxide bonds: A theoretical investigation
Source: Beilstein J Org Chem. 2015 Jul 31;11:1340–51. doi: 10.3762/bjoc.11.144 (PMC4578342; doi:10.3762/bjoc.11.144)
Supplement: File 1 — Atomic coordinates, calculated bond lengths and bond angles as well as calculated Mulliken charges for the reactant state, the precursor state, the transition state and the product state. [file Beilstein_J_Org_Chem-11-1340-s001.pdf]

Supporting Information

for

Surprisingly facile CO_2 insertion into cobalt alkoxide bonds: A theoretical investigation

Willem K. Offermans[1], Claudia Bizzarri[1], Walter Leitner[2] and Thomas E. Müller*[1]

Address: [1]CAT Catalytic Center, RWTH Aachen University, Worringerweg 2, 52074 Aachen, Germany and [2]Lehrstuhl für Technische Chemie und Petrolchemie, ITMC, RWTH Aachen University, Worringerweg 1, D-52074 Aachen, Germany

Email: Thomas E. Müller - thomas.mueller@catalyticcenter.rwth-aachen.de

*Corresponding author

Atomic coordinates, calculated bond lengths and bond angles as well as calculated Mulliken charges for the reactant state, the precursor state, the transition state and the product state.

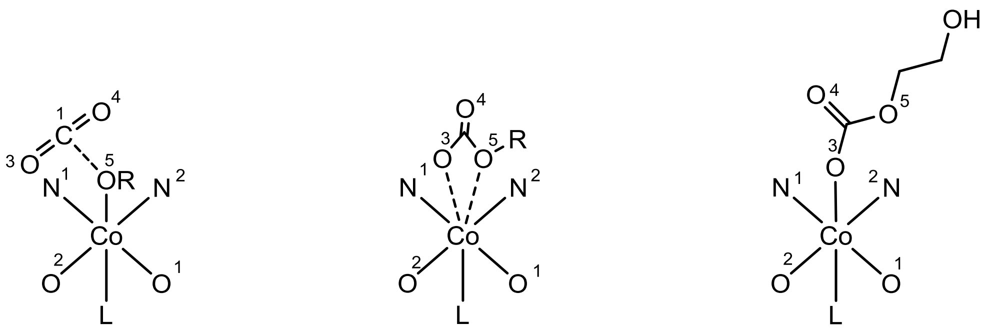

Reactant / precursor state   Transition state   Product state

Scheme S1: Numbering applied in the [Co(Salen)(L)] complexes.

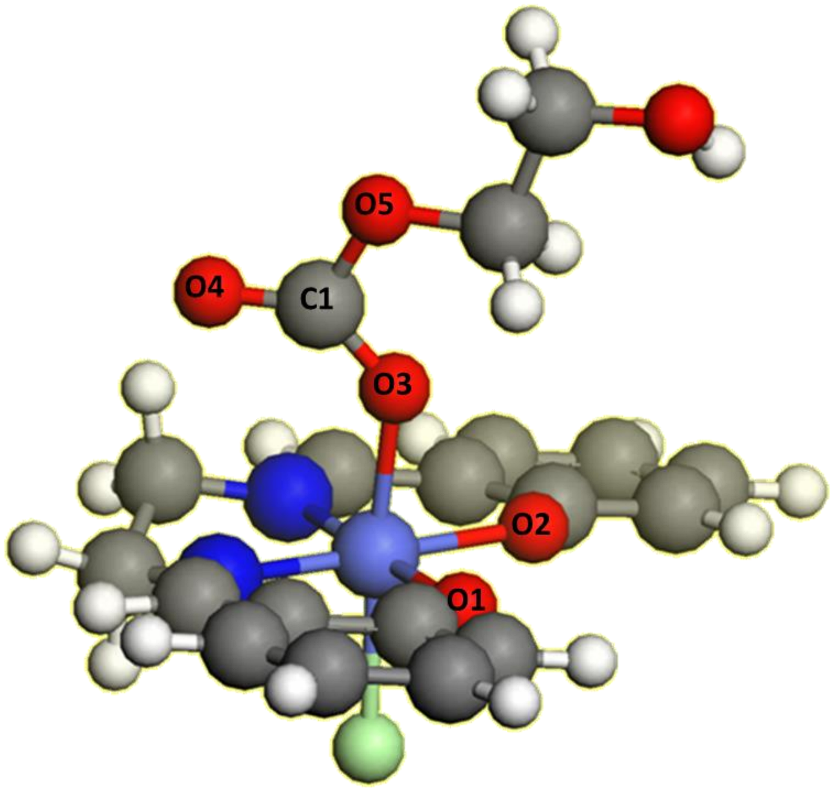

Figure S1: Graphical representation of the product state (**PS**).

Table S1: Calculated bond lengths and bond angles for selected bonds of the reactant state **RS** and calculated Mulliken charge on the cobalt center.

	Bond length [Å]						Angle [°]	Charge† [C]
Ligand	Co-L	Co-OR	Co-N^1	Co-N^2	Co-O^1	Co-O^2	O-Co-OC	Co
Chloride	2.345	1.942	1.912	1.913	1.938	1.932		0.639
CH_3COO^-	1.991	1.933	1.917	1.904	1.940	1.937		0.703
CH_3COO^- [a]	1.995	1.933	1.917	1.908	1.943	1.937		0.700
CH_3COO^- [b]	1.990	1.932	1.912	1.897	1.960	1.961	106.365	0.703
p-Methoxyphenolate	1.983	1.937	1.920	1.919	1.928	1.933	87.439	0.709
CCl_3COO^-	2.034	1.914	1.919	1.906	1.936	1.935	81.459	0.702
2,4-Dinitrophenolate	2.027	1.901	1.921	1.919	1.926	1.926	61.274	0.701
2,4,6-Trinitrophenolate	2.134	1.897	1.924	1.911	1.920	1.916	88.632	0.710
TBD [c]	2.046	1.896	1.923	1.919	1.934	1.957	68.717	0.723

† Mulliken charge

[a] Salen ligand **1a** with cyclohexyl backbone

[b] Salen ligand **1b** with cyclohexyl backbone and *t*-Bu-groups

[c] TBD = 1,5,7-Triazabicyclo[4.4.0]dec-5-ene

Table S2: Calculated bond lengths and bond angles for selected bonds of the precursor state **preS** and calculated Mulliken charge on the cobalt center.

	Bond length [Å]									Angle [°]	Charge† [C]
Ligand	Co-L	Co-OR	Co-N^{1}	Co-N^{2}	Co-O^{1}	Co-O^{2}	O^{5}-CO_2	C-O^{3}	C-O^{4}	∠ (CO_2)	Co†
Chloride	2.278	2.084	1.903	1.922	1.934	1.922	1.678	1.224	1.222	140.054	0.645
CH_3COO^-	1.988	1.942	1.914	1.904	1.941	1.937	2.730	1.176	1.179	172.829	0.708
CH_3COO^- [a]	1.991	1.938	1.919	1.908	1.939	1.937	2.761	1.175	1.179	173.374	0.703
CH_3COO^- [b]	1.986	1.938	1.915	1.902	1.957	1.957	2.783	1.175	1.179	173.838	---
p-Methoxy-phenolate	1.981	1.948	1.918	1.917	1.931	1.932	2.721	1.175	1.179	172.653	0.713
CCl_3COO^-	2.025	1.925	1.918	1.907	1.936	1.936	2.801	1.175	1.178	174.099	0.707
2,4-Dinitro-phenolate	2.020	1.917	1.918	1.920	1.930	1.925	2.801	1.175	1.179	173.704	0.707
2,4,6-Trinitro-phenolate	2.132	1.902	1.922	1.911	1.915	1.923	2.876	1.175	1.178	175.324	0.712
TBD-PL	2.049	1.904	1.921	1.917	1.936	1.955	3.593	1.174	1.176	178.158	0.723
None	---	1.870	1.913	1.898	1.895	1.897	3.034	1.174	1.177	177.663	0.663

† Mulliken charge

[a] Salen ligand 1a with cyclohexyl backbone

[b] Salen ligand 1b with cyclohexyl backbone and *t*-Bu-groups

Table S3: Calculated bond lengths and bond angles for selected bonds of the transition state **TS** and calculated Mulliken charge on the cobalt center.

	Bond length [Å]										Angle [°]	Charge† [C]
Ligand	Co-L	Co-O^5R	Co-O^3	Co-N^1	Co-N^2	Co-O^1	Co-O^2	O^5-CO_2	C-O^3	C-O^4	∠ (CO_2)	Co†
Chloride	2.235	2.927	3.019	1.899	1.908	1.912	1.907	1.434	1.263	1.246	110.206	
CH_3COO^-	1.905	2.860	2.980	1.904	1.894	1.926	1.917	1.441	1.264	1.243	109.909	0.724
CH_3COO^- [a]	1.903	2.950	3.027	1.907	1.903	1.923	1.914	1.438	1.265	1.243	110.090	0.719
CH_3COO^- [b]	1.905	3.471	3.367	1.898	1.888	1.923	1.921	1.446	1.261	1.246	111.184	0.708
p-Methoxy-phenolate	1.930	2.478	2.846	1.918	1.915	1.932	1.923	1.428	1.261	1.242	111.145	
CCl_3COO^-	1.967	3.469	3.196	1.903	1.899	1.912	1.908	1.419	1.263	1.250	112.261	
2,4-Dinitro-phenolate	1.930	2.677	2.880	1.911	1.917	1.915	1.914	1.431	1.265	1.241	109.410	0.729
2,4,6-Trinitro-phenolate	1.984	2.822	2.994	1.912	1.909	1.909	1.900	1.421	1.265	1.245	110.685	0.723
TBD-PL	1.943	2.592	2.850	1.917	1.910	1.939	1.931	1.451	1.261	1.238	109.054	
None	---	2.478	2.846	1.890	1.888	1.890	1.886	1.428	1.261	1.242	111.145	0.640

† Mulliken charge

[a] Salen ligand **1a** with cyclohexyl backbone

[b] Salen ligand **1b** with cyclohexyl backbone and *t*-Bu-groups

Table S4: Calculated bond lengths and bond angles for selected bonds of the product state **PS** and calculated Mulliken charge on the cobalt center.

	Bond length [Å]									Angle [°]	Charge† [C]
Ligand	Co-L	Co-O^3	Co-N^1	Co-N^2	Co-O^1	Co-O^2	C-O^5	C-O^3	C-O^4	∠ (CO_2)	Co†
Chloride	2.286	1.978	1.907	1.915	1.939	1.932	1.411	1.286	1.235	112.371	0.624
CH_3COO^-	1.954	1.977	1.923	1.912	1.936	1.932	1.390	1.292	1.238	113.775	0.706
CH_3COO^- [a]	1.951	1.978	1.915	1.926	1.933	1.932	1.390	1.292	1.238	113.766	0.703
CH_3COO^- [b]	1.954	1.978	1.919	1.909	1.945	1.944	1.396	1.289	1.238	114.216	0.707
p-Methoxyphenolate	1.949	1.985	1.929	1.927	1.917	1.926	1.393	1.291	1.238	113.612	0.715
CCl_3COO^-	1.989	1.954	1.926	1.914	1.928	1.926	1.383	1.298	1.236	113.917	0.704
2,4-Dinitrophenolate	1.973	1.937	1.908	1.920	1.934	1.924	1.401	1.294	1.231	112.119	0.699
2,4,6-Trinitro-phenolate	2.082	1.931	1.931	1.919	1.908	1.913	1.372	1.304	1.234	114.338	0.710
TBD-PL	1.999	1.941	1.932	1.927	1.930	1.948	1.390	1.298	1.233	114.283	0.727
None	---	1.895	1.918	1.903	1.891	1.893	1.365	1.315	1.228	113.944	0.668

† Mulliken charge

[a] Salen ligand **1a** with cyclohexyl backbone

[b] Salen ligand **1b** with cyclohexyl backbone and *t*-Bu-groups
